# Intestinal Absorption and Antioxidant Activity of Grape Pomace Polyphenols

**DOI:** 10.3390/nu10050588

**Published:** 2018-05-09

**Authors:** Veronica Sanda Chedea, Laurentiu Mihai Palade, Daniela Eliza Marin, Rodica Stefania Pelmus, Mihaela Habeanu, Mircea Catalin Rotar, Mihail Alexandru Gras, Gina Cecilia Pistol, Ionelia Taranu

**Affiliations:** Laboratory of Animal Biology, National Research and Development Institute for Biology and Animal Nutrition, Baloteşti (INCDBNA-IBNA), Calea Bucuresti nr. 1, Balotesti, 077015 Ilfov, Romania; mihai.palade@ibna.ro (L.M.P.); daniela.marin@ibna.ro (D.E.M.);pelmus_rodica_stefania@yahoo.com (R.S.P.); mihaela.habeanu@ibna.ro (M.H.); rotar.mircea.catalin@gmail.com (M.C.R.); mihai.gras@ibna.ro (M.A.G.); gina.pistol@ibna.ro (G.C.P.); ionelia.taranu@ibna.ro (I.T.)

**Keywords:** polyphenols, absorption, IPEC-1 cells, piglets, UV-Vis spectra, procyanidin trimers, antioxidant activity

## Abstract

The absorption and antioxidant activity of polyphenols from grape pomace (GP) are important aspects of its valorization as a feed additive in the diet of weaned piglets. This study aimed to evaluate the presence of polyphenols from GP both *in vitro* in IPEC cells and *in vivo* in the duodenum and colon of piglets fed with diets containing or not 5% GP and also to compare and correlate the aspects of their *in vitro* and *in vivo* absorption. Total polyphenolic content (TPC) and antioxidant status (TAS, CAT, SOD and GPx enzyme activity, and lipid peroxidation-TBARS level) were assessed in duodenum and colon of piglets fed or not a diet with 5% GP. The results of UV-Vis spectroscopy demonstrated that in cellular and extracellular medium the GP polyphenols were oxidized (between λmax = 276 nm and λmax = 627.0 nm) with the formation of *o*-quinones and dimers. LC-MS analysis indicated a procyanidin trimer possibly C2, and a procyanidin dimer as the major polyphenols identified in GP, 12.8% of the procyanidin trimer and 23% of the procyanidin dimer respectively being also found in the compound feed. Procyanidin trimer C2 is the compound accumulated in duodenum, 73% of it being found in the colon of control piglets, and 62.5% in the colon of GP piglets. Correlations exist between the *in vitro* and *in vivo* investigations regarding the qualitative evaluation of GP polyphenols in the cells (λmax at 287.1 nm) and in the gut (λmax at 287.5 nm), as oxidated metabolic products. Beside the presence of polyphenols metabolites this study shows also the presence of the unmetabolized procyanidin trimers in duodenum and colon tissue, an important point in evaluating the benefic actions of these molecules at intestinal level. Moreover the *in vivo* study shows that a 5% GP in piglet’s diet increased the total antioxidant status (TAS) and decreased lipid peroxidantion (TBARS) in both duodenum and colon, and increased SOD activity in duodenum and CAT and GPx activity in colon. These parameters are modulated by the different polyphenols absorbed, mainly by the procyanidin trimers and catechin on one side and the polyphenols metabolites on the other side.

## 1. Introduction

Grape pomace (GP) is a waste product from wine making and consists mainly of grape skin residues, broken cells with pulp remains, stalks, and seeds [1,2]. It has been described that grape pomace contains a great amount of polyphenols from the classes of anthocyanins, catechins, flavonols, alcohols, stilbenes, benzoic (gallic, protocatechuic, 4-hydroxybenzoic) and cinnamic (*p*-coumaric) acids [2,3,4]. Polyphenols are bioactive compounds having many health benefits, due to their antioxidant, anticancer, antifungal and antibacterial properties [5]. In farm animal, for example in pig, the polyphenols derived from grape marc extract exerted anti-inflammatory effect in the small intestine, which is particularly important for this species [6]. At weaning, the young pig is subjected to a myriad of stressors (e.g., change in nutrition, separation from mother and littermates, new environment etc.) which cause reduced growth [7] and predispose animals to inflammation and diseases. Significant changes in the histology and biochemistry of the small intestine, such as villous atrophy and crypt hyperplasia were associated with weaning that could cause the decrease of digestive and absorptive capacity and contribute to post-weaning diarrhea [7]. This post-weaning ‘growth check’ continues to represent a major source of production loss in many commercial piggeries [7]. Nutritive bioactive components such as polyphenols from plant and food industry by-products are promising sources to counteract these symptoms.

As with all bioactive substances, the beneficial actions of polyphenols in animals are largely dependent on their bioavailability at the target tissue [8,9]. This bioavailability depends on polyphenols’ absorption and metabolism at the gastrointestinal tract as well as on tissue metabolism and cellular distribution after absorption [9]. In this context there is considerable interest in clarifying the bioavailability of these components of the diet and their bioactivity *in vivo* [9,10]*.* There is considerable controversy surrounding the current studies on the absorption and metabolism of polyphenols and results are therefore inconclusive [9,11]. Studies on absorption are rendered difficult by the molecular complexity of the extracts or polyphenol-rich feed owing to factors like their level of polymerization and conjugation with other phenols [9,11]. Most polyphenols are present in food in the form of esters, glycosides or polymers that cannot be absorbed in their native form [9,11]. These substances must be hydrolyzed by endogenous enzymes or microbiota before they can be absorbed [9,11]. Once absorbed, polyphenols are recognized by the body as xenobiotics, and their bioavailability is therefore relatively low in comparison to micro- and macronutrients [9,11]. The metabolization of polyphenols takes place through a sequence of reactions common to all of them. This is similar to a metabolic detoxication to reduce their potential cytotoxic effect by increasing their hydrophilicity and facilitating urinary or biliary elimination [9,12].

The aim of this study was to evaluate the presence and absorption of polyphenols derived from GP, used as a beneficial dietary alternative source of natural compounds, *in vitro* on IPEC-1 cells as well as *in vivo*, in duodenum and colon of piglets during the post weaning period. Due to the limitation of *in vivo* testing, in order to check the absorption and bioavailability of nutrients and bioactive compounds, cell models are gaining a growing interest among the scientific research investigations [13]. *In vitro* studies may offer a suitable alternative for in vivo animal testing being representative of the *in vivo* physiology [13]. Cell culture models can support massive screening and cost effectiveness in contrast to the more expensive animal trials with limited screening capacity [13]. Out of all the animal-derived models obtained, the pig intestinal cell model is of interest and is being increasingly used in *in vitro* studies on absorption and bioavailability of nutrients and bioactive compounds. In this work the *in vitro* study on intestinal porcine epithelial cells was also used to compare and check the correlation existing between the *in vitro* and *in vivo* assessment of polyphenols absorption by UV-Vis spectroscopy.

As mentioned the absorption of polyphenols derived from GP was also assessed *in vivo*, in the duodenum and colon of weaned piglets fed a diet with 5% GP. Piglets performance, total polyphenol content (TPC), lipid peroxidation assessed by TBARS test, total antioxidant status (TAS) and antioxidant enzymes’ activity, catalase (CAT), superoxide dismutase (SOD) and glutathione peroxidase (GPx), were also determined *in vivo*.

Pig was also regarded as an experimental model, being considered to be the most suitable non-primate animal model for nutrition studies, due to the high degree of similarity between pig and human to digestion, anatomy and physiology of the gastrointestinal tract [14,15], the composition of the intestinal microbiota being very similar [16]. Due to these similarities, the results of polyphenols absorption and their activity in pigs might also be applied to human nutrition [16]. Also, due to its similarity with the human gut, it represents a good platform for the simulation of various human intestinal applications [13,17].

## 2. Materials and Methods

### 2.1. Aqueous GP Extraction and Total Polyphenols Content Determination

The GP was provided by Dionis Ltd. (Târgovişte, Romania), a Romanian producer of grape seed oil and derived from red varieties of grapes from Valea Calugărească, a Romanian winery. The pomace consisting of stems, skins and seeds was dried in a heated air flow. GP raw material was milled to a particle size of less than 6 mm in a Cyclone Mill-MC5 (Tecator, Höganäs, Sweden) and incorporated in the conventional feed compound in a proportion of 5%.

For the aqueous extraction the ratio of GP powder: hot water (almost at boiling point) was 1:3. The mixture was vortexed for 2 h and then filtered through Whatman filter paper. Aqueous extracted GP (AGP) was then lyophilized and kept at room temperature. For further *in vitro* analyses the lyophilized AGP was reconstituted in water. 

Total polyphenols content was determined as described before [4]. The results were expressed as mg gallic acid equivalents (GAE)/100 g dry GP.

#### UV-Vis Spectroscopy of AGP Extract

The spectrum was recorded at room temperature using a spectrophotometer (Specord 250, Analytik Jena, Jena, Germany) in the UV-Vis range 250–750 nm [18].

### 2.2. In Vitro Study

#### 2.2.1. Measurement of Cell Viability (MTT Assay)

Intestinal porcine epithelial cell line (IPEC-1) derived from the small intestine of newborn non-suckled piglets was kindly provided by Dr. P. Pinton, Laboratory of Toxicology-Pharmacology, INRA, Toulouse, France.

Cell viability in response to grape pomace extract was assessed through MTT assay as described by Marin et al. (2011) [19]. Briefly, 2 × 10^5^ IPEC-1 cells/mL were seeded in DMEM F12 culture media in 96 well plates, incubated at 37 °C until they reached 80% confluence (2–3 days) and then treated with different concentrations of AGP extract (250 ng GAE/mL, 500 ng GAE/mL, 1000 ng GAE/mL, 2500 ng GAE/mL and 5000 ng GAE/mL AGP). After 24 h incubation with AGP, 10 μL MTT solution in PBS (5 mg/mL) was added to each well and mixed thoroughly. After a further 2 h incubation at 37 °C, 10 μL of MTT solvent (0.1 N HCl in anhydrous isopropanol) was added to each well and plates were read within 1 h of MTT solvent addition. The absorbance was measured at 570 nm using a microplate reader (TECAN SUNRISE, Salzburg, Austria) and the absorbance of the background at 650 nm was subtracted. All tests were performed in four independent experiments.

#### 2.2.2. Cells Treatment and UV-VIS Spectra Measurement

IPEC-1 cells were seeded at 2 × 10^5^/mL in DMEM F12 culture media in 6 well plates and incubated at 37 °C until confluence. Then, cells were incubated with 250, 500, 1000 ng GAE/mL AGP for 3 h or 24 h. After the incubation with the AGP extract, the cells were washed with HBSS (Hank’s Balanced Salt Solution) and treated with trypsin for the cell detachment. The cell suspension was then centrifuged and the pellet washed twice in HBSS. The cell pellet was then suspended in 10 μL water. 

The spectra were recorded at room temperature using a spectrophotometer (Specord 250, Analytik Jena, Jena, Germany) in the UV-Vis range 250–750 nm [18].

The UV-Vis spectra were recorded from the collected extracellular medium after a 3 h treatment period (EST) and a 24 h treatment period (ELT) of AGP co-incubation with the cells. The cell sediment suspended in water represents the cellular matrix was also collected after 3 h (CST) and 24 h (CLT) of treatment with AGP. The blank for spectra measurement was the correspondent untreated control cells of each experimental sample.

After registering the UV-Vis spectra, from each treatment variant spectrum the correspondent untreated cells’ variant spectrum was subtracted and the λmax of the remaining spectra were determined, using the software of the spectrophotometer. These subtractions were overlayed using the Overlay function of the apparatus [18].

### 2.3. In Vivo Study

#### 2.3.1. Animals and Diets

For this study, a total number of 20 crossbred TOPIG hybrid [(Landrace × Large White) × (Duroc × Pietrain)] pigs with an average body weight of 10.70 ± 0.8 kg were allocated to two experimental groups (10 pigs/group). The animals individually identified by ear tag were housed in pens (5 pigs/pen) and fed with isoenergetic and isoproteic experimental diets containing 5% grape pomace (experimental group, GP+) or 0% GP (control group, GP−) for 36 days (Table 1).

Dry matter (DM), crude protein (CP), fat (EE), crude fibres (CF) and ash of basal and experimental diets as well as of grape pomace were determined according to the ISO methods (ASRO-SR EN ISO, 2010) and consist in 88.87% (GP−), 88.90% (GP+) and 87.63% (GP) for DM; 18.53% (GP−), 18.71% (GP+) and 10.32% (GP) for CP; 2.18% (GP−), 2.70% (GP+) and 5.14 % (GP) for EE; 4.22% (GP−), 5.36% (GP+) and 25.01% (GP) for CF; 6.12% (GP−), 5.19% (GP+) and 5.75% (GP) for ash.

The grape pomace was provided by a local producer and derived from Valea Calugarească, a Romanian winery. The pomace consisting of stems, skins and seeds was dried in a heated air flow. GP raw material was milled to a particle size of less than 6 mm in a Cyclone Mill-MC5 (Tecator, Höganäs, Sweden) and incorporated in the conventional feed compound in a proportion of 5% (Table 1). Pigs were individually weighed at the beginning, after 15 days and at the end of the trial and feed intake was recorded daily per pen. The average daily gain (ADG), average daily feed intake (ADFI), and feed—gain ratio (F/G) were calculated for the periods 1–15 days, 16–36 days and 1–36 days. Pigs had free access to water and assigned diet during the 36-day experimental period. The animals were cared for in accordance with the Romanian Law 43/2014 for handling and protection of animals used for experimental purposes and the EU Council Directive 98/58/EC concerning the protection of farmed animals. The study protocol was approved by the Ethical Committee of the National Research-Development Institute for Animal Nutrition and Biology, Balotesti, Romania (Ethical Committee no. 52/2014, approval no. 3220/27.05.2016). All animals remained healthy during the experimental period and no veterinary drugs were used. At the end of the experiment (day 36), animals were slaughtered by exsanguination in an EU-licensed abattoir according with the EU Council directive 2010/63/CE.

#### 2.3.2. Sample Preparation

At the end of the experiment (36 days) animals were slaughtered and samples of duodenum and colon from animals in both groups were collected. The samples were kept at −80 °C until analysis (UV-Vis spectra and LC-MS). Aliquots from frozen tissue samples were milled in liquid nitrogen (IKA works, 2900000 A_11_ basic Analytical Mill) and the resulted powder was used for analysis.

#### 2.3.3. Polyphenols Extraction from GP and Experimental Diets (GP− and GP+)

One gram of GP or feed compound (GP− and GP+) (Table 1) was mixed with 2 mL acidic (1% HCl) methanol solution, vortexed for 30 s, sonicated 30 min and then centrifuged for 10 min at 3000 rpm/min. The supernatants representing the extracts (MGP, GP− and GP+) were collected and stored at −20 °C for further analyses. 

#### 2.3.4. Polyphenols Extraction from Duodenum and Colon Samples

Polyphenols from organs were extracted with methanol 100%. One gram of frozen powder sample of duodenum, control (D−) and the treated samples (D+), and colon, control (C−) and experimental samples (C+) was mixed with 10 mL MetOH (100%) and crushed with an Ultra Turrax (Jahnke and Kunkel, IKA, Staufen, Germany) [20]. The mixture was treated for 30 min in an ultrasonic water bath at 4 °C and then centrifuged for 10 min. The supernatant representing the methanolic extracts of D−, D+, C− and C+ respectively was kept at −20 °C until further analyses.

#### 2.3.5. Measurement of Polyphenols Composition in GP, Diets and Gut Samples by High Performance Liquid Chromatography-Photodiode Detector Coupled with Mass Spectroscopy (HPLC-DAD-MS)

The composition of polyphenols in methanolic extracts (GP, GP−, GP+, D−, D+, C− and C+) was determined by HPLC-DAD-MS based on their retention times, UV-Vis spectra (200 to 600 nm) and the mass spectrum of individual compounds using standard compounds according to the method of Dulf et al. [21] with slight modifications. Prior to LC-MS analyses the extracts were filtered through 0.45 μm syringe filters (Whatman, Bucharest, Romania). An Eclipse, XDB C18 column was used [(4.6 × 150 mm, 5 μm); (Agilent Technologies, Paolo Alto, CA, USA)]. The mobile phases gradient consisted of 0.1% acetic acid in distilled water (v/v) (solvent A) and 0.1% acetic acid in acetonitrile (v/v) (solvent B) at a flow rate of 0.5 mL/min for 30 min using the following gradient elution program: 0 to 2 m (5% B), 2 to 18 m (5 to 40% B), 18 to 20 m (40 to 90% B), 20 to 24 m (90% B), 24 to 25 m (90 to 5% B) and 25 to 30 m (5% B).

The catechins and derivatives were detected at 280 nm and the anthocyanins at 520 nm. Data analysis was performed using Agilent ChemStation Software (Rev B.04.02 SP1, Paolo Alto, CA, USA). The catechins and derivatives were calculated as equivalents of catechin (mg catechin/100 g DW of substrate; r^2^ = 0.9985). Anthocyanin levels were determined using cyanidin chloride as an external standard and expressed as equivalents of cyanidin (mg cyanidin/100 g DW of substrate) (r^2^ = 0.9951).

#### 2.3.6. Measurement of UV-VIS Spectra from Duodenum and Colon Samples

The spectra for duodenum and colon samples were recorded at room temperature using a spectrophotometer (Specord 250, Analytik Jena, Jena, Germany) in the UV-Vis range 250–1050 nm [18].

After registering the UV-Vis spectra of all samples (D−, D+, C− and C+) the control spectra, D− and C− respectively, were subtracted from each D+ and C+ spectrum, the λmax of the remaining spectra was determined, using the software of the spectrophotometer. These subtractions were overlayed using the Overlay function of the apparatus [18].

#### 2.3.7. Determination of the Total Phenolic Content from Duodenum and Colon

Total phenolic content (TPC) was determined using the Folin–Ciocalteu method [22] adapted to a microscale [23] as follows: 40 μL of organs extracts were mixed with 1560 μL of distilled water, plus 100 μL of Folin–Ciocalteu reagent and vigorously stirred. After exactly 1 min, 300 μL of aqueous sodium carbonate (20%) was added, and the mixture vigorously stirred again and then allowed to stand at room temperature, in the dark, for 90 min. The absorbance was then read at 750 nm (on a UV-visible diode array spectrophotometer Specord 250, Analytik Jena, Jena, Germany) and TPC was calculated from a calibration curve, using gallic acid as standard. The results were expressed as mg gallic acid equivalents (mg GAE)/100 g tissue.

#### 2.3.8. Preparation of Tissue (Duodenum and Colon) Lysate

Frozen tissue samples (200 mg) were disrupted and homogenized by using Ultra-Turrax homogenizer (IKA-Werke GmbH & Co. KG, Staufen, Germany) and 50 mM potassium phosphate (pH 7.0, containing 1 mM EDTA per gram tissue) for CAT, 20 mM HEPES buffer (pH 7.2, containing 1 mM EGTA, 210 mM mannitol and 70 mM sucrose per gram tissue) for SOD, 50 mM Tis-HCl (pH 7.5, containing 5 mM EDTA and 1 mM DTT per gram tissue) for GPx and 1 × PBS (EDTA free) for TAS and TBARS were added. The homogenates were kept 30 min on ice, and then centrifuged at 10,000× *g* at 4 °C for 10 min. If not assayed immediately, the supernatants (tissue lysates) were frozen at −80 °C (stable for 1 month).

#### 2.3.9. Measurement of Lipid Peroxidation in Duodenum and Colon—TBARS-MDA

Lipid peroxidation was analysed as previously described by Ohkawa et al. (1979) [24]. The mixture of reaction contained 10 µL of sample (appropriately diluted tissue lysate), 240 µL of deionized water, 25 µL of 0.5 N HCl and 250 µL thiobarbituric acid (TBA). The mixture was incubated at 95 °C for 15 min and the reaction was stopped by an immediate transfer of each tube to ice. Subsequently, the reaction mixture was added to a microplate well and measured in fluorescence mode (exc. 515 nm; em. 548 nm). The results were expressed as nmol MDA/g tissue, using 1,1,3,3,-tetramethoxypropane (TMP) as standard.

#### 2.3.10. Measurement of Total Antioxidant Status (TAS) in Duodenum and Colon

Antioxidant capacity of tissue samples was measured with the TEAC (Trolox equivalent antioxidant capacity) assay using ABTS as radical cation (2,2′-azinobis-(3-ethylbenzothiazoline-6-sulphonic acid), method adapted by the one described by Miller et al. (1993) [25]. Briefly, 30 µL ABTS, 10 µL metmyoglobin and 127 µL of buffer (of which 10 µL were replaced with the appropriately diluted sample which was being investigated) were mixed and then incubated at 37 °C for 3 min. The absorbance was measured at 732 nm. The reaction was then initiated by the addition of 33 µL of hydrogen peroxide. Spectra were recorded again at 732 nm. The results were expressed as mmol/L Trolox equivalents (plasma) and µmol TE/g of tissue (organ tissue). 

#### 2.3.11. Measurement of Catalase (CAT) Activity in Duodenum and Colon

Catalase activity was measured using Catalase Assay Kit (Cayman Chemical) according to the manufacturer’s instructions. The method is based on the reaction of an enzyme with methanol in the presence of optimal H_2_O_2_ concentration. The produced formaldehyde is measured colorimetrically with 4-amino-3-hydrazino-5-mercapto-1,2,4-triazole (Purpald) as chromogen. Purpald specifically forms a bicyclic heterocycle with aldehydes, which upon oxidation, changes from colourless to purple. Briefly, 100 μL of diluted Assay Buffer, 30 μL of methanol, and 20 μL of sample (appropriately diluted tissue lysate) were mixed with 20 μL of hydrogen peroxide in each well of a 96 wells plate; the plate was then covered and incubated on a shaker for 20 min at room temperature. 30 μL of potassium hydroxide was added were used to stop the reaction. After a 10-min incubation on the shaker with Catalase Purpald (30 μL per well), the solution was finally incubated with potassium periodate (5 min, 10 μL per well). Absorbance was read at 540 nm using a microplate reader (Tecan Infinite M200, Salzburg, Austria). Results (CAT activity) were expressed as µmol/min/g tissue. One unit was defined as the amount of enzyme that will cause the formation of 1.0 nmol of formaldehyde per minute at 25 °C.

#### 2.3.12. Measurement of Superoxide Dismutase (SOD) Activity in Duodenum and Colon

Superoxide Dismutase activity was measured by using SOD Cayman Assay Kit according to the manufacturer’s instructions. The assay allows the measurement of all three types of SOD enzymes and uses tetrazolium salt for detection of superoxide radicals generated by hypoxanthine and Xanthine Oxidase. Briefly, 200 μL of diluted Radical Detector and 10 μL of sample (appropriately diluted tissue lysate) were mixed and the reaction was initiated by adding 20 μL of diluted Xanthine Oxidase to each well. Then, the plate was covered and incubated on a shaker for 30 min at room temperature. Absorbance was read at 440–460 nm with a microplate reader (Tecan Infinite M200). Results (SOD activity) were expressed as U/g tissue. One unit was defined as the amount of enzyme needed to exhibit 50% dismutation of the superoxide radical. SOD activity is standardized using the cytochrome c and xanthine oxidase coupled assay.

#### 2.3.13. Measurement of Glutathione Peroxidase (GPx) Activity in Duodenum and Colon

A Glutathione Peroxidase Assay Kit manufactured by Cayman Chemical was used. Employing this method, the GPx activity is indirectly measured by a coupled reaction with GR (glutathione reductase) through which the oxidation of NADPH to NADP+ is translated by a decrease in the absorbance measured at 340 nm. Briefly, 100 μL of final Assay Buffer, 50 μL of GPx Co-Substrate Mixture, and 20 μL of sample (appropriately diluted tissue lysate) were added to each well of a 96 well plate (Greiner^®^, Bucharest, Romania). The reaction was initiated by the addition of 20 μL of GPx Cumene Hydroperoxide to each well and the plate was carefully mixed for a few seconds. The absorbance was read every minute at 340 nm with a plate reader (Tecan Infinite M200). Results (GPx activity) were expressed as µmol/min/g tissue. One unit was defined as the amount of enzyme that will cause the oxidation of 1.0 nmol of NADPH to NADP+ per minute at 25 °C.

### 2.4. Statistical analysis

The results were presented as mean values ± standard errors of the mean (SEM) from at least three independent measurements. Each pig was considered an experimental unit. Data were analysed with StatView software 6.0, SAS Institute, Cary, NC, USA performing one-way analysis of variance (ANOVA), followed by a Fisher protected least significant difference (PSLD) test. *p*-Values lower than 0.05 were considered significant while *p* values between 0.05 and 0.1 were considered as tendencies.

## 3. Results

### 3.1. In Vitro Study

#### 3.1.1. UV-Vis Spectroscopy Analysis and Total Polyphenols Concentration of AGP Extract

**Figure 1:**
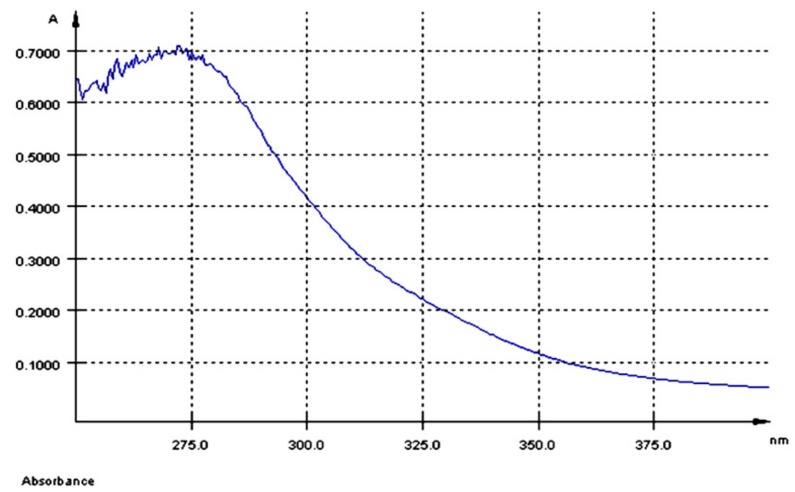
UV-Vis spectrum of the aqueous grape pomace extract (AGP).

The UV-Vis spectrum of AGP extract had a maximum of absorption at λmax = 280 nm (Figure 1) and a total polyphenol content of 5.8 mg GAE/100 g dry GP.

#### 3.1.2. Effect of AGP Extract on Cell Viability

Cell viability in response to AGP was assessed through MTT assay. After 24 h of exposure to the AGP extract, it was found that doses between 250 ng GAE/mL, 500 ng GAE/mL and 1000 ng GAE/mL did not affect the cell viability which remained almost 100% of control (99.08% for 250 ng GAE/mL, 100.45% for 500 ng GAE/mL and 93.69% for 1000 ng GAE/mL).

#### 3.1.3. Evaluation of AGP’s Fate in Extracellular Medium

**Figure 2:**
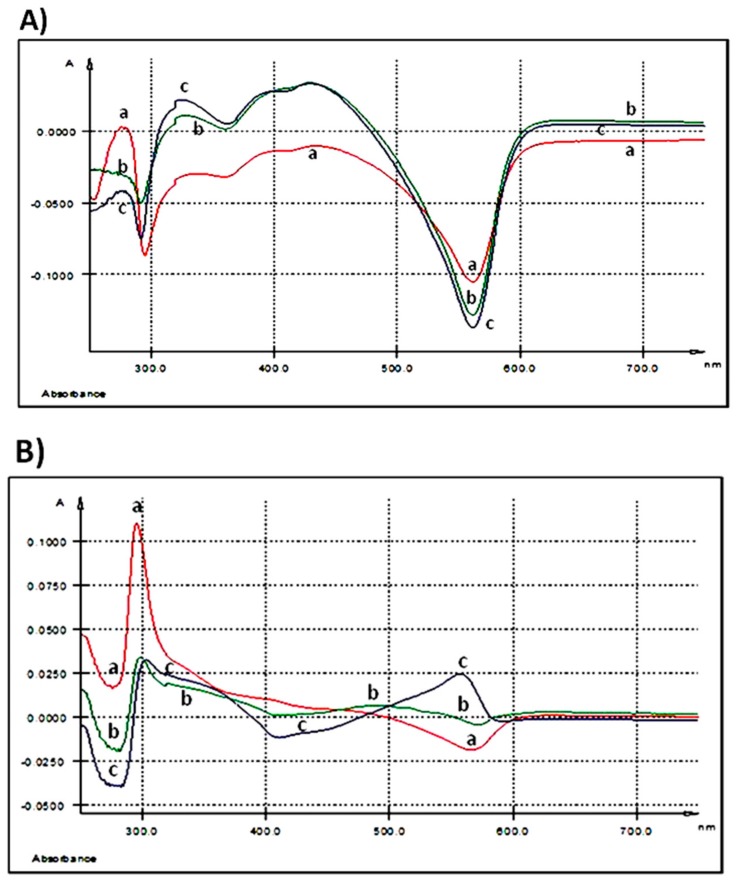

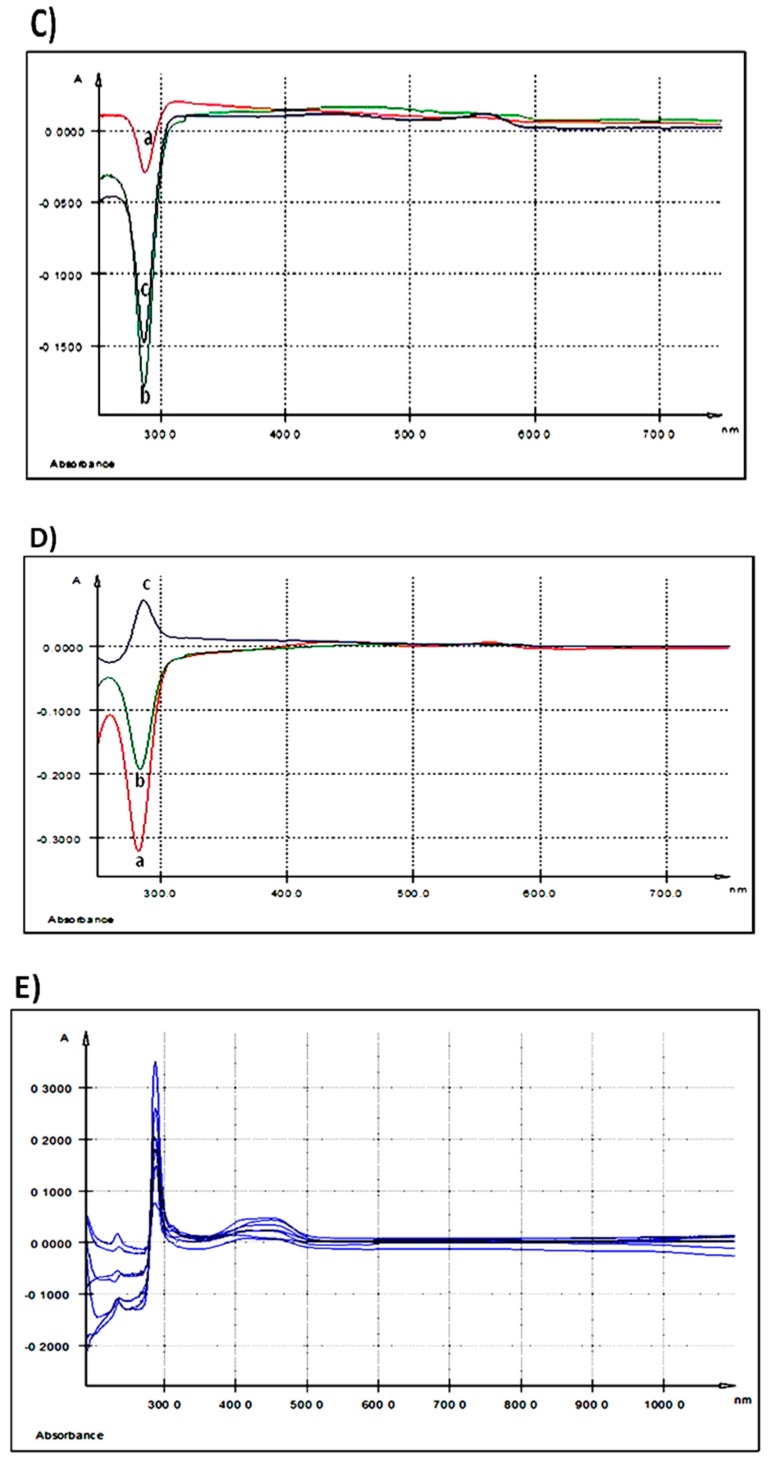

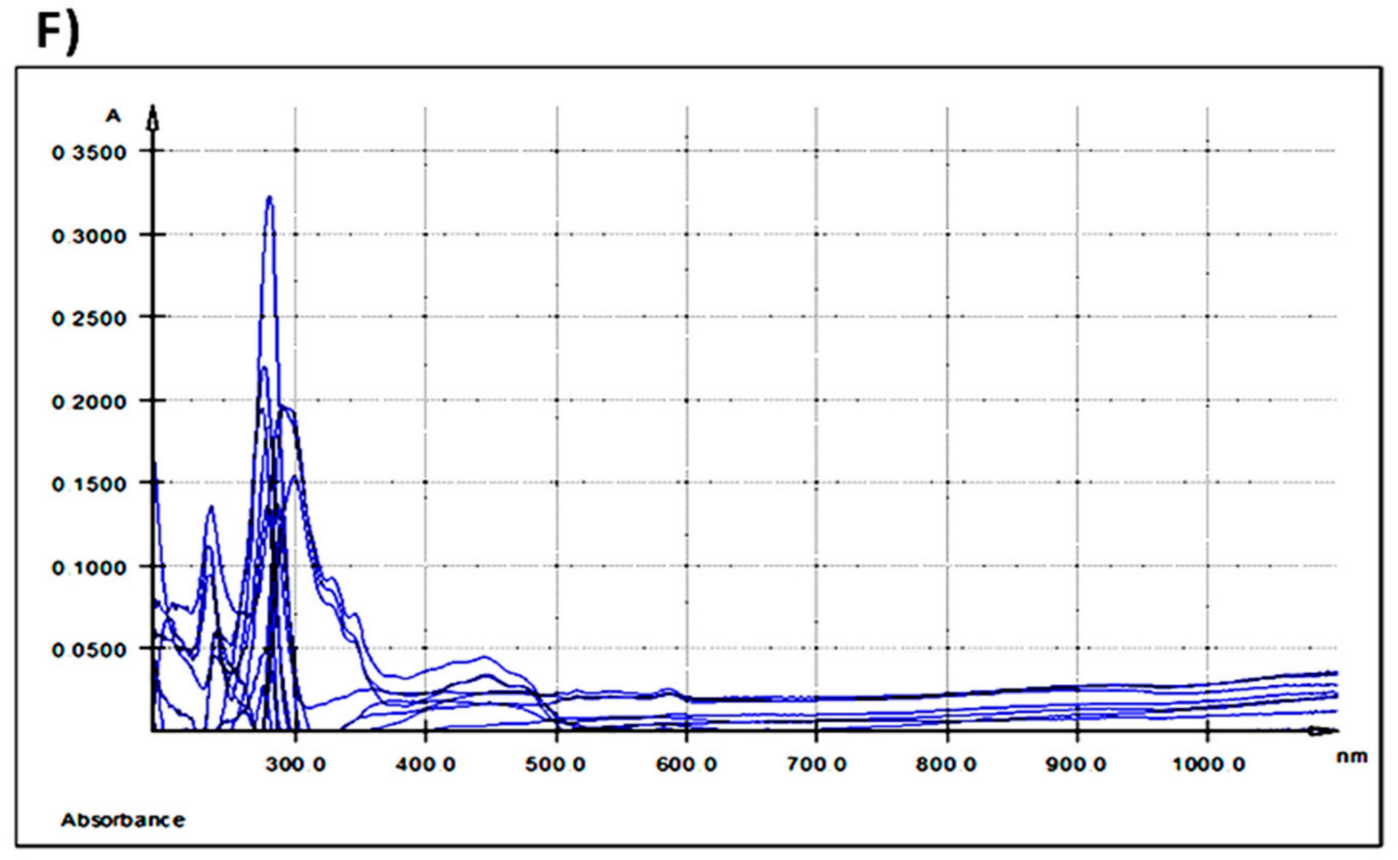
UV-Vis spectra: (**A**) extracellular medium (IPEC-1 cells) after 3 h of treatment with different AGP concentrations; (**B**) extracellular medium (IPEC-1 cells) after 24 h of treatment with different AGP concentrations; (**C**) intracellular matrix (IPEC-1 cells) after 3 h of treatment with different AGP concentrations; (**D**) intracellular matrix (IPEC-1 cells) after 24 h of treatment with different AGP concentrations; (**E**) differences between duodenum samples originating from the experimental (5% GP diet) and the control (no GP diet) groups; (**F**) differences between colon samples originating from the experimental (5% GP diet) and the control (no GP diet) groups; (a-red—250 ng GAE/mL AGP, b-green—500 ng GAE/mL AGP and c-blue—1000 ng GAE/mL AGP).

After 3 h of 250 ng GAE/mL treatment, UV-Vis spectrum of AGP did not show any relevant maximum of absorption (Table 2, Figure 2A).

As the spectrum was registered taking as control the medium of the untreated cells, we can say that the extract kept its initial composition in the extracellular culture medium after 3 h of exposure. When the dose increases at 500 ng GAE/mL two maxima were seen, the first one at λmax = 327.9 nm and the second at λmax = 428.7 nm with a shoulder (sh) at 399.5 nm (Table 2, Figure 2A). Doubling the dose at the maximum concentration tested, 1000 ng GAE/mL, the EST spectrum presents three maxima, one at λmax = 325.3 nm, the second at λmax = 393.3 nm and the third at λmax = 427.6 nm (Table 2, Figure 2A).

A prominent absorption peak (λmax = 295.6 nm) (Table 2 and Figure 2B) was observed in the extracellular medium after 24 h of treatment when the IPEC-1 cells were exposed to the lowest concentration (250 ng GAE/mL). At 500 ng GAE/mL, the absorption peak decreased in intensity. The treatment of the cells with the highest dose of 1000 ng GAE/mL determines a maximum absorption at λmax = 557.5 nm (Table 2 and Figure 2B).

#### 3.1.4. Evaluation of AGP’ Fate in the Cellular Matrix

No relevant absorption peaks were registered in cellular matrix of IPEC-1 cells after 3 h of co-incubation with all the three AGP concentrations in comparison with untreated cells (Table 2 and Figure 2C). In contrast, incubation with AGP extract for 24 h revealed an effect at the highest dose tested, the most prominent peak was seen at λmax = 287.1 nm (Table 2 and Figure 2D). The absorption maxima for the lowest dose of 250 ng/mL AGP was at λmax = 438.2 nm and λmax = 561.2 nm and for 500 ng GAE/mL AGP at λmax = 579.3 nm (Table 2 and Figure 2D).

### 3.2. In Vivo Study

#### 3.2.1. Polyphenol Content and Profile of GP and Feed Compounds

**Figure 3:**
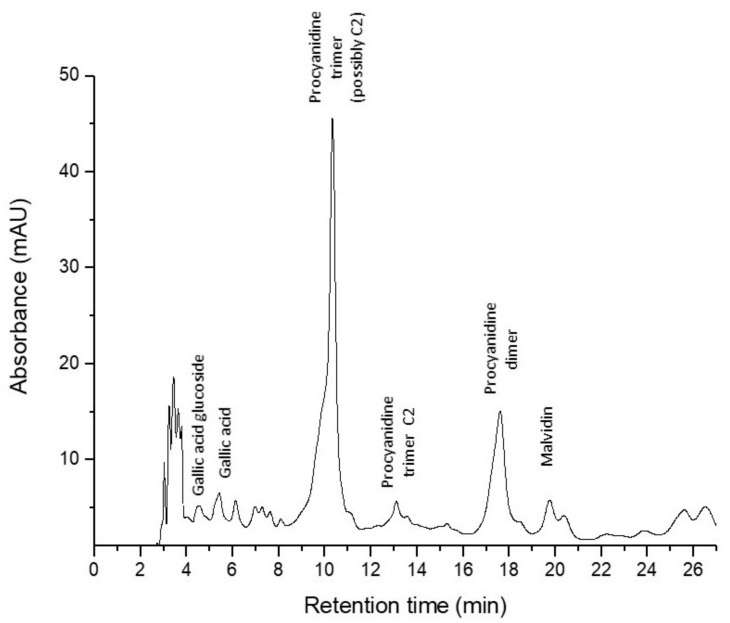
Grape pomace chromatogram.

The qualitative and quantitative analysis of polyphenols’ content of GP and of feed compounds, was determined by HPLC-DAD-MS-ESI+ based on their retention times, UV-visible spectra and the mass spectra of individual compounds using standard compounds and literature data (Table 3). Figure 3 presents the chromatogram of GP extract.

In GP a procyanidin trimer (possibly C2) was found in the highest amount (16.54 mg CE/100 g), followed by a procyanidin dimer and equally by gallic acid-glucoside, gallic acid, and a procyanidin trimer C1 (Table 3). Malvidin 3-*O*-(6″-coumaroyl-glucoside) was found in the lowest concentration in GP (Table 3). Galic acid, ferulic acid derivate (ferulic acid dehydrotrimer), caffeoylquinic acid, daidzin (daidzein-7-*O*-glucoside), *p*-coumaroylquinic acid, 6″-*O*-malonyldaidzin, genistin (genistein-7-*O*-glucoside), ferulic acid, 6″-*O*-malonylgenistin, dicaffeoylquinic acid were eluted and identified in equal amounts in both feed samples, GP− as well as GP+ (Table 3). Procyanidin trimer (possibly C2) (2.118 mg CE/100 g) and procyanidin dimer (1.803 mg CE/100 g) were found in small amounts only in the experimental feed (GP+) with 5% GP (Table 3).

#### 3.2.2. Effect of Dietary GP on the Growth Performance of Piglets

The effects of GP+ diet on growth performance of piglets are presented in Table 4. After 15 days of GP+ feeding, no significant differences in ADG, ADFI and F/G were observed among treatments. From days 16 to 36, or days 1 to 36, there were no significant differences in ADG, and F/G among groups, although a slight increase in total ADG was registered in piglets fed GP+ diet (613.89 g vs. 570.99 g). However, a significant increase for ADFI was noticed when GP was administrated (1255.14 g vs. 1097.33 g).

#### 3.2.3. Qualitative Assessment of Polyphenols Absorption in Duodenum and Colon by UV-Vis Spectra Measurement

Table 5 shows all the absorption maxima registered for duodenum and colon samples. The overlaid UV-Vis spectra of duodenum methanolic extracts, indicated two absorption bands. The first band, band I, had a maximum of absorption at λmax = 287.5 nm and the second band, band II, at λmax = 430 nm (Table 5 and Figure 2E).

The UV-Vis spectra of colon samples reveal four absorption bands: band I having the absorption maxima at λmax = 279 nm, band II at λmax = 292 nm, the most intense one, band III showing the λmax at 444 nm and the fourth band, band IV, having λmax at 587 nm (Table 5 and Figure 2F).

#### 3.2.4. Quantitative Assessment of Polyphenols Absorption in Duodenum and Colon by LC-MS Measurement

A procyanidin trimer (C2) was identified by LC-MS in both control and experimental duodenum samples, but higher amounts were found in the samples derived from the piglets fed the 5% GP diet (16.00 mg CE/100 g) than in the samples taken from the piglets receiving the control diet (13.69 mg CE/100 g) (Table 6).

In contrast, in colon samples, besides the aforementioned procyanidin trimer (C2) present in the duodenum, another two polyphenols, catechin and procyanidin trimer C1 were also found in both control and GP+ group (Table 6). The first procyanidin trimer eluting at t_R_ = 10.16 min was present in equal amounts in the colon of piglets from both groups, catechin and procyanidin trimer C1 were slightly higher in colon derived from C− group than in colon derived from C+ (9.45 vs. 8.73 for catechin and 6.85 vs. 5.38 for procyanidin C1, respectively) (Table 6).

#### 3.2.5. Total Polyphenols Content in Duodenum and Colon

**Figure 4:**
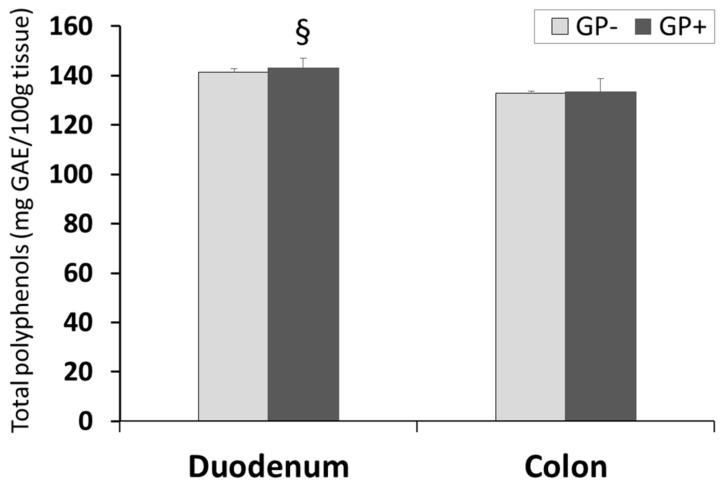
Total polyphenols (mg GAE/100 g tissue) as determined by the Folin–Ciocalteu method, in duodenum and colon samples (n = 10); § = statistically significant compared to colon GP+.

The results presented in Figure 4 showed that there were no differences concerning the TPC level in duodenum and colon of pigs fed with or without GP diet. However, the concentration of TPC was significantly higher in duodenum than in colon tissue irrespective of the diet (GP+ or GP−, Figure 4). 

#### 3.2.6. Effect of GP+ Diet on Lipid Peroxidation (TBARS-MDA)

**Figure 5:**
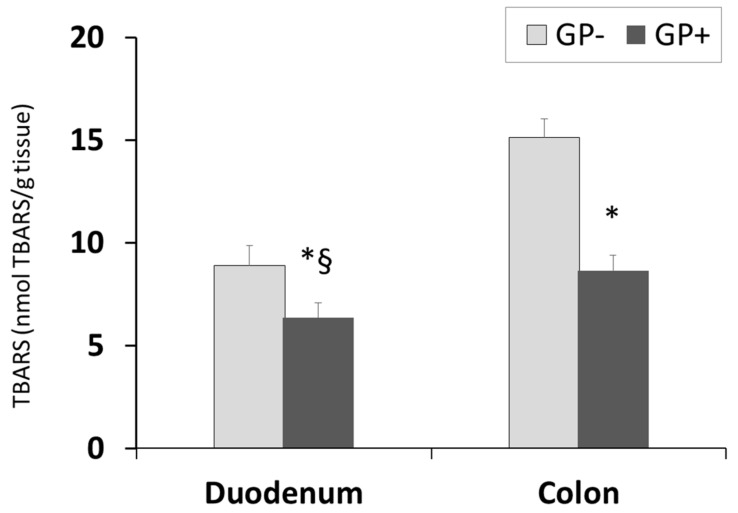
Lipid peroxidation (nmol TBARS/g tissue) as determined by the TBARS assay, in duodenum and colon samples (n = 10); * = statistically significant when GP− was compared with GP+, § = statistically significant when compared with colon GP+.

The diet including 5% of GP was able to significantly decrease the lipid peroxidation in the duodenum and colon of piglets fed GP+ diet (Figure 5). TBARS-MDA levels decreased by 29% in duodenum and 43% in the colon. Conversely to the aforementioned parameter, the TBARS concentration was lower in the duodenum than in the colon irrespective of the diet.

#### 3.2.7. Effect of GP+ Diet on Total Antioxidant Status (TAS)

**Figure 6:**
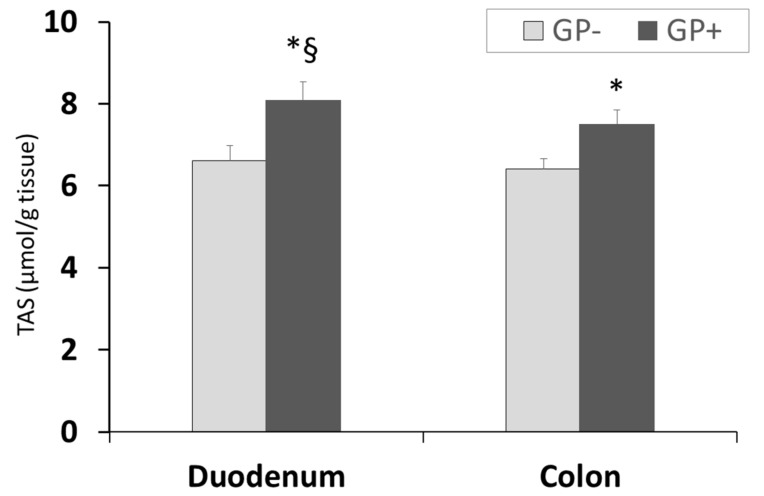
Total antioxidant status (µmol/g tissue) as determined by the TAS assay, in duodenum and colon samples (n = 10); * = statistically significant when GP− was compared with GP+, § statistically significant when compared with colon GP+.

Assessment of TAS showed a higher antioxidant status/capacity in duodenum compared with colon and for both organs a significant (*p* < 0.05) increase was registered in the case of GP+ diet compared with control (8.10 μmol/g tissue GP+ vs. 6.60 μmol/g tissue GP− for duodenum and 7.50 μmol/g tissue GP+ vs. 6.40 μmol/g tissue GP− for colon, Figure 6).

#### 3.2.8. Effect of Antioxidant Enzymes Activity

**Figure 7:**
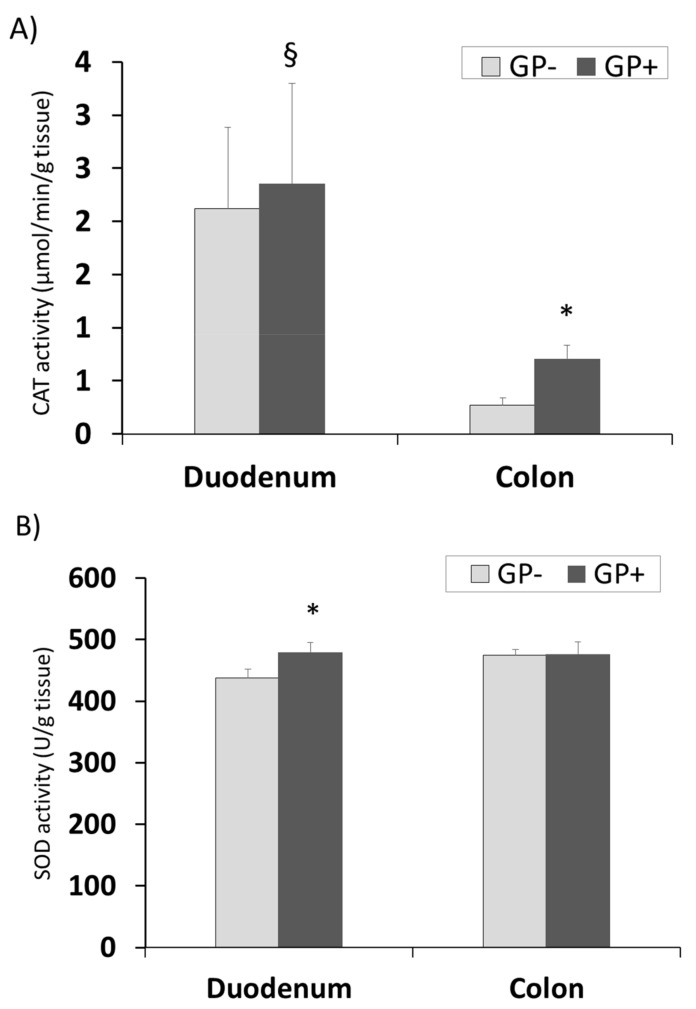

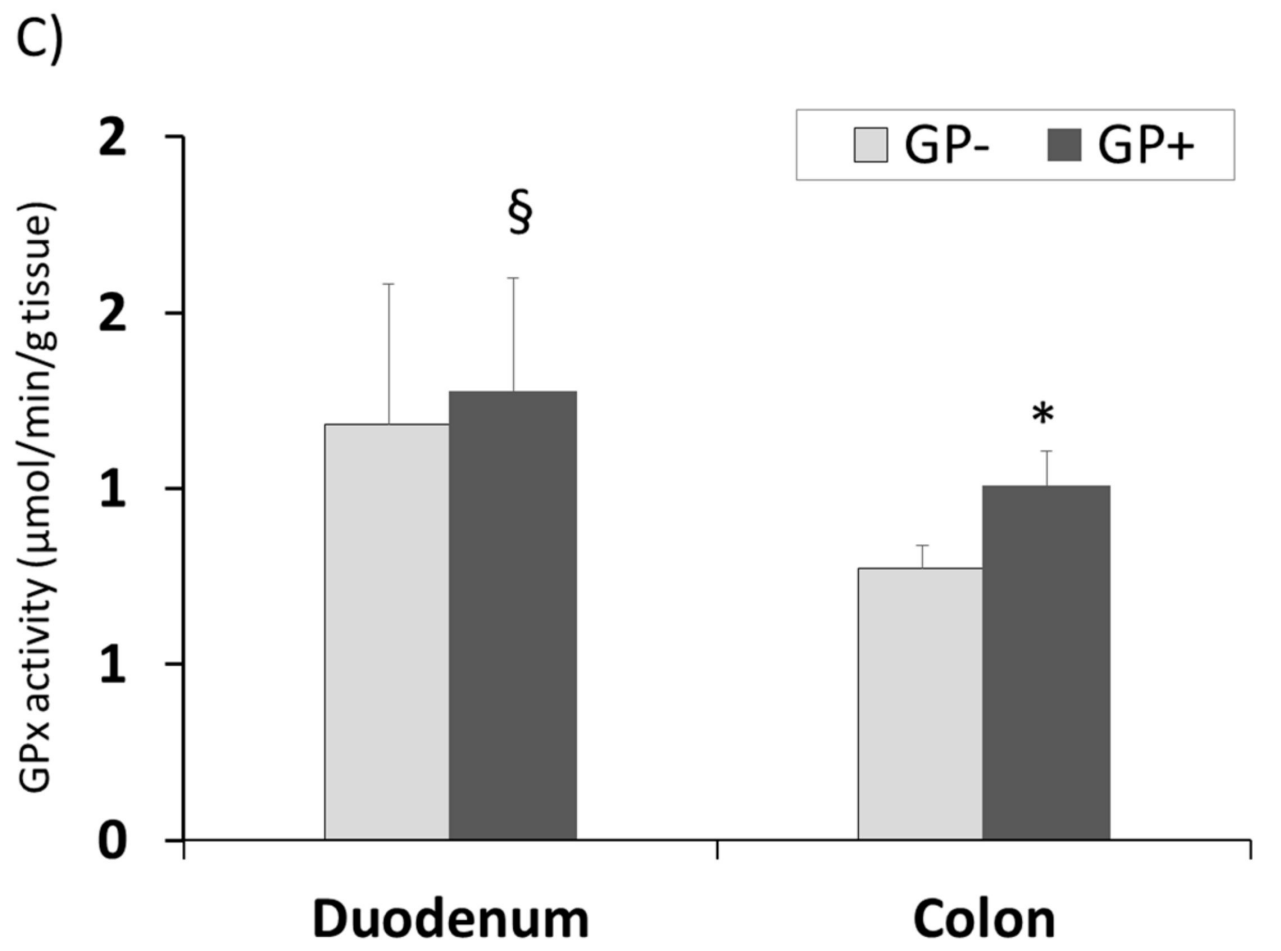
Antioxidant activity in duodenum and colon samples (n = 10) as determined by: catalase (CAT) activity (µmol/min/g tissue) (**A**), superoxide dismutase (SOD) activity (U/g tissue) (**B**), glutathione peroxidase (GPx) activity (µmol/min/g tissue) (**C**); * = statistically significant when GP− was compared with GP+, § = statistically significant when compared with colon GP+.

The same tendency for CAT and GPx activity with higher concentrations in duodenum compared with colon was registered. Also a significantly higher level was found in the colon samples derived from the piglets fed 5% GP (Figure 7A,C). SOD activity remained unchanged in colon tissue irrespective of the diet but increased significantly in duodenum of pigs fed GP+ diet (+9%, Figure 7B).

## 4. Discussion

Polyphenols are considered as promising feed additives in the nutrition of farm animals based on the fact that conditions of oxidative stress and inflammation are highly relevant in farm animals, especially in pigs during weaning period [6]. In contrast to many studies performed with animal and human models, the potential antioxidative and anti-inflammatory effects of polyphenols on farm animals have been less investigated so far [6]. In pigs, besides several diseases associated with systemic inflammation, the weaning phase represents an important stage in which a local inflammation in the small intestine occurs, called weaning-associated intestinal inflammation [6,26,27,28]. It has been originally suggested that these effects are mainly due to a depressed feed intake and to a still undeveloped enzymatic equipment [6,26,27]. Adverse effects of weaning stress on gut function are not limited to the weaning phase, rather, weaning stress triggers long-term defects in intestinal barrier function [6,29]. Therefore, the weaning-associated intestinal inflammation process is negatively linked with health and growth performance of piglets, and controlling this process is a challenge to managing post-weaning health and optimum growth performance [6,28] especially after the banning of antibiotics (2006). Nutritional interventions based on feed bioactive compounds are very promising and therefore investigated. In this direction, in the *in vivo* experiment of present study, pigs after weaning were fed with diets containing or not 5% dried grape pomace and the results showed an increase in average daily feed intake (ADFI) without significantly affecting the body weight in the case of GP diet. This result is close to the result reported by Hao et al., (2015) who found for weaned piglets (6.99 ± 0.11 kg body weight) that dietary supplementation with grape seed procyanidins did not exert significant effects on growth performance (ADG, ADFI and F/G) during an overall experimental period of 28 days [30]. Other studies confirm also that feeding diets containing plant polyphenols did not affect the piglets growing performances [31], but exert a positive effects on different aspects of health, antioxidant status and inflammation.

The benefic actions of red GP are due to its rich composition in phenolics: hydroxycinnamic acids (C6–C3) gallic acid and protocatechuic acid, flavonoids including anthocyanins [3] and flavanols (catechin, epicatechin, gallocatechins) [4,32] plus other active compounds (fibers, PUFA etc.). In addition, oligomers (from 2 to 5 units) and polymers of flavanols are in relevant concentrations, with a significant predominance of type-B procyanidins [32]. Because, oligomers and polymers with low levels of solubility are not extracted during winemaking processes and remain in the wine pomace [32], GP is a good source of these compounds and through dietary intake their presence might be expected at tissue level. In our study we found also a high level of procyanidins in the used GP.

In view of its benefic actions, we assessed in this study the fate of a GP extract rich in procyanidins on *in vitro* intestinal epithelial cells IPEC-1 as well as its absorption and antioxidant actions at intestinal level, *in vivo* in weaned piglets as a possible way of preventing the post-weaning problems. Taking into consideration the literature evidences which show that the plasma concentrations in intact flavonoids rarely exceed 1μM and that the maintenance of a high plasma polyphenol concentration requires repeated ingestion, in the *in vitro* experiments of the present study we used low graded dietary levels of GP extract such as 250 ng GAE/mL (1.46 μM), 500 ng GAE/mL (2.93 μM) and 1000 ng GAE/mL (5.87 μM) in order to measure *in vitro* the maximum absorption in intestinal cells [11,12]. A bathochromic shift of λmax—indicating an oxidation of aqueous grape pomace (AGP) polyphenols—has been observed after 3 h of treatment with the higher AGP concentrations added to cell culture media. Also, a longer incubation time (24 h) of AGP with IPEC-1 cells resulted in a high UV-Vis absorption with maximum at λmax = 295.6 nm in the extracellular medium for the lowest AGP concentration administrated. This result is in accordance with our previous study [18] in which the UV-Vis measurement showed that a grape seed extract and a pure catechin was oxidized in primary leucocyte culture (bathocromic shift from λmax = 280 nm to λmax = 308 nm for the extract and to λmax = 297.5 for pure catechin respectively). In a study concerning the EGCG (epigallocatechin gallate) stability in culture media it was observed that this flavan-3-ol was unstable in McCoy’s 5A culture medium, in the presence or the absence of HT-29 human colon adenocarcinoma cells, with the formation of dimers and with a display of an increased absorbance over a broad range of 350–400 nm [33]. Several factors, including pH, concentration of proteins, antioxidant levels, and the presence of metal ions, could affect the stability of EGCG, of which pH is probably the most critical [33]. Dangles et al. indicated that for catechin the oxidation takes place with the formation of a catechin o-quinone and an absorption maximum at 335 nm that then evolves into yellow dimers absorbing in the region 400–500 nm [34]. Our results were similar with these observations, λmax in the extracellular medium for both incubation times and for all the AGP doses tested being in the range 327.9–557.5 nm. 

In our *in vitro* study, after 3 h of AGP treatment no effect was seen, but IPEC-1 cells incubated with AGP extract for 24 h gave the best absorption of polyphenols from the extract at the highest administrated concentration (1000 ng GAE/mL); this result was not obtained for the lower concentrations (250 ng GAE/mL and 500 ng GAE/mL). The maximum absorption (λmax) in this case was at 287.1 nm which was higher than that of AGP extract (270 nm). This bathochromic shift may indicate, like in the extracellular medium, that a slight oxidation of polyphenolic molecules has occurred within the cells.

As a general trend we hypothesized that in both cellular or extracellular medium the polyphenols from AGP underwent an oxidative process which started with a maximum of absorption at λmax = 276.0 nm and ended with λmax = 627.0 nm; based on our previous study [18] in which for λmax between 393.3 nm and 487.5 nm (451.5 nm, 438.2 nm, 434 nm, 487.5 nm, 428.7 nm sh at 399.5 nm, 393.3 nm, 427.6 nm) we identified *o*-quinones as the oxidation products of AGP polyphenols. The same authors [35] reported further an LC-MS analysis evidence attesting the involvement and fate of quercetin-quinone in the pro-oxidant behaviour of a quercetin and caffeic acid mixture. Besides their antioxidant properties, catechins have been described as displaying pro-oxidant activity, having the potential to be oxidized to o-quinones or semiquinones, which leads to redox cycling and reactive oxygen species production as well as in thiol, DNA and protein alkylation [36,37]. Because flavonoids tend to act as antioxidants rather than oxidants, at a first glance, oxidative stress generated by the production of quinones does not seem relevant to explain the curative properties of these compounds. It was shown that to some extent the grape seed extract, considered as an antioxidant nutritive supplement, might have pro-oxidant activity as well, depending on dose, duration of administration and other dietary components [18]. The UV-Vis analysis proved that the antioxidant activity of this extract might be mediated by the pro-oxidant quinones/oxidation products of the polyphenols from grape seeds [18].

It has been recognized in the recent years that low levels of oxidants have physiological functions for stress adaptation [6]. Thus, physiological level of oxidants are even useful for the adaptation of the body to cellular stress due to the improving defense and detoxification mechanisms [6]. In line with this, it has been proven that moderate production of ROS in the mitochondria improves health and even extends the life span of different model organisms (*C. elegans* and mice), a phenomenon that has been named mitochondrial hormesis or abbreviated mitohormesis [6,38]. 

In order to check the fate of the dietary GP at the intestinal level of weaned piglets, the composition in polyphenols of GP, GP+ and GP− feed has been analyzed by LC-MS. The results indicated a procyanidin trimer possibly C2, and a procyanidin dimer as the major polyphenols identified in GP (Table 2). These procyanidins were present also in the ingested GP+ feed, 12.8% for the procyanidin trimer and 23% for the procyanidin dimer respectively. In both GP− and GP+ diet the caffeoylquinic acids, ferulic acid and the isoflavones: daidzin, 6″-*O*-malonyldaidzin, genistin and 6″-*O*-malonylgenistin were identified in equal amounts. 

From all the groups of polyphenols identified in GP and GP+ diet, only the procyanidin trimer C2 seemed to be accumulated in slightly higher concentration in the duodenum (14.4%) derived from piglets receiving this diet for 36 days. Because polyphenols bioactivity is mostly altered by conjugation and methylation, the conserved activity of the free procyanidin dimers and trimers *in vivo* might partly compensate their lower absorption compared with monomers like catechin and epicatechin [39]. Furthermore, bioavailability of procyanidins might be underestimated, because synergy between low- and high-degree of polymerization oligomers seems to take place. Because procyanidin contents of foods are relatively high, procyanidin trimers might contribute to a protective effect of polyphenol-rich foods. 

Also, studies of [40] showed that in the case of dietary matrices rich in polyphenols like catechins and procyanidins, these compounds pass through the gastrointestinal tract almost unmodified. Procyanidins with a low grade of polymerization (dimers and trimers) were detected in the intestine and plasma samples [41]. It has been estimated that 90–95% of ingested procyanidins enter the colon unaltered along with some unabsorbed monomers [42]. In colon, procyanidins and their monomeric units (epi)catechin are catabolized by colonic microflora into a series of low molecular weight phenolic acids and the health potential of these metabolites should be studied as the original procyanidins presented in plasma in only low amounts [39].

In our study, 73% of the procyanidin trimer C2 identified in duodenum was found in the colon of control piglets, and 62.5% respectively in the colon of pigs fed 5% GP diet. Besides this compound the colon tissue samples of piglets from both groups contained catechin and procyanidin trimer C1 with lower amounts for C+ than for C− (Table 3). This result indicates a different absorption of different isomers in duodenum and colon irrespective of the concentration based on slight structural differences (procyanidin trimer C1 versus procyanidin trimer C2). 

The LC-MS analysis of the control diet without GP did not revealed the presence of any procyanidin trimers and catechin, but these compounds were identified in the colon of piglets fed control diet. In the case of colon samples (C+) derived from the piglets fed the GP+ diet, catechin might resulted by the decomposition of the procyanidin dimer and trimer C2 identified in GP+ feed which passed from duodenum in colon. 

In an attempt to explain the presence of trimers in colon (C−) and duodenum (D−) samples we evaluated their spectrum by UV-Vis measurement. The modified spectrum detected by UV-Vis indicated the fact that the polyphenols were mainly structurally modified by metabolization and in this form they were absorbed in the duodenum and colon at λmax between 274.9 nm and 298.0 nm. This shift from 280 nm, λmax of AGP, shows a slight oxidation following the ingestion. Like in the case of *in vitro* experiments, the absorption maxima between 315 nm and 588 nm might be explained by and extensive oxidation at inter and intra molecular level between polyphenols and proteins or other antioxidants and molecules with the formation of *o*-quinones and dimers-possibly trimers, as indicated by the LC-MS results. 

Regarding the higher catechin and procyanidine trimer C1 colonic uptake in GP− samples, some assumptions were made in the view of the literature data. Recently it was suggested that the oral bioavailability of bioactive components could be increased by a food excipient particularly containing phytochemicals that can regulate their bioaccessibility, absorption, and transformation [43,44]. For instance, quercetin altered the methylation of catechins by binding catechol-O-methyltransferase (COMT) due to its strong binding affinity to COMT [44]. It was also reported that quercetin was shown to act as an efflux inhibitor, proposing that co-ingestion of green tea with quercetin could increase the bioavailability of green tea catechins [44]. Piperine from black pepper improved the absorption of EGCG and steamed rice up-regulated the transporter MRP in epithelial cells to increase the concentration of EGC from green tea in plasma of mice [44]. Co-incubation of milk with green tea enhanced the transepithelial absorption of green tea catechins in Caco-2 cell [44]. 

In this context the presence of the other polyphenols in feed could influence the absorption of catechin and procyanidin trimer C1 in function of their binding affinity to catechol-O-methyltransferase (COMT). Biotransformation of catechins such as methylation by COMT occurs in the small intestine [44,45,46]. The greater susceptibility of flavonols over other flavonoids to methylation in the intestinal membrane may exist in the specificity of COMT for these compounds [44,47]. If the catechins have a stronger binding affinity to COMT than the dietary bisoflavones daidzein and genistein, than a less amount of catechins will be absorbed when both classes of polyphenols are ingested. Indeed methylated forms of the catechol metabolites, which were generated by incubations of the soy isoflavones daidzein and genistein with COMT *in vitro* could be detected only in trace amounts in the human urine samples [48]. This implies that this reaction does not play a major role in the biotransformation of the hydroxylated daidzein and genistein metabolites *in vivo* [48]. 

The differences in the qualitative and quantitative absorption of catechins in duodenum and colon might be given also by the modulation of gut microbiota by these compounds. The gut microbiota metabolize unabsorbed procyanidins to smaller phenolic compounds which are highly bioavailable [42]. Grape compounds have also demonstrated the ability to increase the abundance of beneficial bacterial species, including *Bifidobacterium* and *Lactobacillus* spp. in animal models [42,49]. These changes could alter overall host health status, but the impacts of dose and compound structure also must be considered [42,49]. 

A good health status is also the result of a well-balanced antioxidant activity level. The antioxidant status of the duodenum and colon of piglets fed or not with the 5% GP diet as well as the TPC was also assessed. Epidemiological, clinical and nutritional studies of Arenas et al., Torabian et al., Baroudi et al., in humans, analyzing the TPC content mainly in plasma and urine after the intake of a diet rich in polyphenols showed an increased TPC [50,51,52,53] or no difference [54] comparing with the control group. In plasma of cows fed a diet with 15% grape pomace TPC increased with 35% compare to control cows [4]. In piglets, this study showed that the diet including GP did not have any effect on TPC in duodenum and colon and this might be due to the low percentage of GP in feed, 5%, compared with 15% in the case of cows diet from our previous study [4]. Baroudi et al. (2013) indicated that the different bioavailability of polyphenols is the reason of not ensuring a high TPC in plasma [54]. 

GP diet increased TAS in both the colon and duodenum of piglets and this result is better than those of antioxidant capacity testing in the plasma of piglets fed grape seed and grape mark diet [55] or with plant extracts including grape seeds [31]. Also, Taranu et al. (2018) registered no different TSA in comparison with the control in the liver of fattening pigs fed grape seed diet [56]. 

TBARS-MDA is the main by-product of lipid peroxidation, and previous studies reported that the antioxidant compounds (flavonoids, phenols, PUFA) from grape extracts or grape by-products are able to control and diminish the TBARS level by their ability to scavenge free radicals [56,57,58]. 

Many studies provided evidence about the reducing effect of dietary polyphenols on TBARS-MDA in plasma or liver but very few on duodenum or colon. For example, administration of grape seed extract inhibited MDA formation in the liver tissues of ICR/f rat and in the body and liver of rabbit [56,59]. In weaned piglets, the study of Zhang et al. (2014) showed that supplementation of the diet with a combination of plant extracts including grape seed (2 g/kg) was effective in reducing TBARS formation in the plasma [31]. Grape seed and grape marc did not influence TBARS concentration in plasma and liver of piglets [55] and in the liver of fattening pigs fed grape seed diet [56].

The intestinal tract is one of the most vulnerable tissues to oxidative stress [60]. The exposure to luminal ROS is an on-going process and it is due to oxidized food debris, high levels of iron ions, saliva oxidants, toxins, bacteria and bile acids [60]. This is highly relevant to the colon, where residence time of luminal contents is prolonged. Indeed, pathological disorders associated with ROS, such as inflammation and cancer, are more common to the colon than to other parts of the gut [60]. In order to cope with the high exposure to oxidative stress, the gut mucosa, like other tissues, possesses several ‘lines of defense’ against ROS [60]. These include repair mechanisms, prevention of the ROS production cascade (e.g., by antioxidant bioactive compounds like polyphenols) and the antioxidants defense system including the antioxidant enzymes, such as SOD, GPx and CAT, the main parameters involved in the cellular defense against free radicals [56,60]. Herein, independently of the diet, duodenum exhibited a higher level of CAT and GPx activity compared to colon. The involvement of ROS in colon diseases such as colitis and cancer [1,2,8,9,10,11,12,13] and the lower susceptibility of the upper part of the GI tract to inflammatory diseases may be partially due to differences in the antioxidant profiles and enzymes activity of these tissues. Addressing the regional differences in antioxidant enzymes’ activities in the rat intestine, Moghadasian and Godin (1996) reported that while glutathione levels were significantly higher in the mucosa of the rat small intestine compared to the mucosa of the colon, no differences were found in the levels of GPx and SOD of these segments [61,62]. In contrast, Siegers and coworkers found a gradual decrease in the activity of GPx from the proximal small intestine of the rat towards the colon [63]. Pig fed GP+ diet produced a higher activity of the three antioxidant enzymes in duodenum, which was significant for SOD. Very important was the fact that in colon the activity of these two enzymes (CAT and GPx) was significantly increased by GP+ diet compared to control. This results confirmed the results showing the beneficial effect of antioxidant compounds in intestine and especially in colon. 

In this respect, in the present study the inclusion of 5% GP in piglets’ diet shows an increase of CAT and GPx activity in colon, a positive aspect in preventing the pathological disorders associated with ROS, such as inflammation and cancer, more common to this segment of the intestinal tract. 

## 5. Conclusions

*In vitro* incubation of the grape pomace extract with IPEC-1 cells showed that the highest absorption of polyphenols in the cells was produced at 1 μM, the highest concentration tested and with the longest treatment period. The UV-Vis spectra of the duodenum methanolic extract derived from pigs fed *in vivo* with GP+ diet had the same absorption maximum, λmax = 287 nm like *in vitro* in IPEC-1 cells. The UV-Vis measurements showed that there were structural changes of the ingested polyphenols as the absorption maxima indicated the formation of oxidation products. Taken together, these *in vitro* and *in vivo* results showed that between these two types of methodologies there are positive correlations concerning the qualitative evaluation of polyphenols in the cells and in the gut.

The presence of polyphenols in duodenum and colon was proven further by the LC-MS analysis. This analysis highlighted the absorption of the unmetabolized procyanidin trimers in duodenum and colon, which might be an important point in evaluating the action of these molecules at intestinal level. Even in low concentrations they determined important benefic changes in the antioxidant status at the intestinal level. In both duodenum and colon, the 5% grape pomace diet lead to a decrease of lipid peroxidation and an increase of the total antioxidant status. The antioxidant enzyme activity was different between the two organs. Thus, superoxide dismutase activity increased significantly in duodenum while catalase and glutathione peroxidase activity increased in colon.

## Figures and Tables

**Table 1:** Compound feed and calculated nutrient content of experimental diets (%).

Ingredients (%)	Weaning Phase ^1^	
	Control Diet (Days 1–36)	GP+ Diet (Days 1–36)
Corn	57.32	53.90
Rice meal	-	-
Wheat	10.00	8.00
Sunflower meal (31.94% CP)	5.00	5.00
Soybean meal (44% CP)	16.00	16.00
Sunflower oil	0.20	0.70
Milk powder	3.00	3.00
Gluten	4.00	4.00
Grape pomace	-	5.00
Monocalcium phosphate	1.25	1.25
Limestone	1.56	1.48
NaCl	0.20	0.20
Metionine	0.03	0.05
Lisine	0.34	0.32
Choline	0.10	0.10
Mineral vitamin-premix ^2^	1.00	1.00
Calculated Nutrient content		
Dry matter (%)	88.31	88.01
Crude Protein (%)	18.29	18.36
Digestible crude protein (%)	14.98	15.06
Fat (%)	2.85	2.96
Crude fiber (%)	4.07	5.30
Metabolisable energy (kcal/kg)	3169	3147
Lysine (%)	1.08	1.08
Digestible Lysine (%)	0.92	0.92
Met + Cys (%)	0.65	0.65
Calcium (%)	0.90	0.90
Phosphorus (%)	0.65	0.65
TOTAL	100.00	100.00

^1^ BW range 9.5 to 38.5 kg; ^2^ Vitamin-mineral premix/kg diet: 10,000 UI vit. A; 2000 vit. D; 30 UI vit. E; 2 mg vit. K; 1.96 mg vit. B1; 3.84 mg vit. B2; 14.85 mg pantothenic ac.; 19.2 mg nicotinic ac; 2.94 mg vit. B6; 0.98 mg folic ac.; 0.03 mg vit.B12; 0.06 biotin; 24.5 mg vit.C; 40.3 mg Mn; 100 mg Fe; 100 mg Cu; 100 mg Zn; 0.38 I; 0.23 mg Se.

**Table 2:** Absorption maxima for the overlaid UV-Vis spectra subtractions of IPEC-1 cells’ extracellular medium and cellular matrix treated with AGP (*in vitro* test).

*In Vitro*	3 h			24 h		
	250 ng GAE/mL	500 ng GAE/mL	1000 ng GAE/mL	250 ng GAE/mL	500 ng GAE/mL	1000 ng GAE/mL
Extracellularλmax (nm)	276.0	327.9 428.7 399.5	325.3 393.3 427.6	295.6	298.3 487.5 627.0	303.6557.5
Cellularλmax (nm)	313.1 554.9	451.5 515.1 539.0 581.9	434.0 559.1	438.2 561.2	579.3	287.1

**Table 3:** Phenolic profile and the phenolic compounds concentration (mg CE*/100 g) in GP, GP+ feed and GP− feed samples (LC-MS evaluation).

Compound	R_t_ (min)	UV λmax (nm)	[M − H]^+^	GP mg CE */100 g	GP+ mg CE/100 g	GP− mg CE/100 g
Gallic acid-glucoside	4.29	258	333, *171*	2.22	-	-
Gallic acid	5.06	272	171	2.71	4.61	4.60
Procyanidin trimer (possibly C2)	11.67	280	867, *290*	16.54	2.12	-
Procyanidin trimer C1	13.01	280	867, *290*	2.24	-	-
Procyanidin dimer	17.91	280	579, *290*	7.79	1.80	-
Malvidin (3-*O*-6″-coumaroyl-glucoside)	20.07	275, 532	639, *331*	0.76	-	-
Ferulic acid derivate (Ferulic acid dehydrotrimer)	10.75	324	565, *195*	-	12.92	12.92
Caffeoylquinic acid	12.21	326	355, *181*	-	43.98	43.97
Daidzin (Daidzein-7-*O*-glucoside)	14.87	262	417	-	14.24	14.23
*p*-Coumaroylquinic acid	15.23	316	339, *193*, *165*	-	24.57	24.56
6″-*O*-Malonyldaidzin	16.81	255	503	-	11.88	11.88
Genistin (Genistein-7-*O*-glucoside)	17.11	265	433	-	16.22	16.21
Ferulic acid	17.82	312	195	-	15.33	15.32
6″-*O*-Malonylgenistin	18.81	260	519	-	17.27	17.25
Dicaffeoylquinic acid	20.81	326	517, *355*, *181*	-	18.96	18.96

Grape pomace = GP, feed compound with 5% GP = GP+, control feed compound without GP = GP−. * The amount of each identified compound in 100 g initial sample (grape pomace or feed compounds) was expressed in catechin equivalents excepting malvidin 3-*O*-(6″-coumaroyl-glucoside) which was quantified as cyanidine equivalents.

**Table 4:** Effect of dietary grape pomace diet on the growth performance of piglets.

Growth Performance	GP−		GP+		*p-*Value
	AVRG	SEM	AVRG	SEM	
Body weight (kg) at day 1	10.67	0.300	10.90	0.256	0.600
Body weight (kg) at day 15	17.89	0.655	18.15	0.742	0.797
Body weight (kg) at day 36	31.22	1.188	33.00	1.370	0.346
days 1 to 15					
ADG (g)	481.48	41.61	483.33	40.14	0.975
ADFI (g)	862.80	21.24	898.00	22.02	0.260
F/G	1.92	0.19	1.99	0.19	0.780
days 16 to 36					
ADG (g)	634.92	31.99	707.14	39.53	0.179
ADFI (g)	1264.85 ^a^	51.74	1510.24 ^b^	38.83	0.0005
F/G	2.03	0.10	2.22	0.18	0.371
days 1 to 36					
ADG (g)	570.99	34.29	613.89	35.00	0.395
ADFI (g)	1097.33 ^a^	45.72	1255.14 ^b^	56.45	0.033
F/G	1.98	0.11	2.10	0.16	0.627

GP− = group fed with control diet, GP+ = group fed with 5% dried grape pomace diet, ADG = average daily gain, ADFI = average daily feed intake, F/G = feed/gain ratio. Values represent the mean of n = 10 determinations ± SEM. ^a,b^ Means that do not share the same letter within a raw are significantly different.

**Table 5:** UV-Vis absorption maxima for the overlaid spectra subtractions of duodenum and colon samples of piglets fed with a control or 5% GP diet.

Samples	λmax (nm)			
Duodenum	287.5	-	430	-
Colon	279	292	444	587

**Table 6:** Phenolic profile and phenolic compounds content (mg CE/100 g) in duodenum, (D+ and D−) and colon (C− and C+) samples evaluated by LC-MS.

Compound	R_t_ (min)	UV λmax (nm)	[M − H]^+^	Duodenum mg CE */100 g		Colon mg CE */100 g	
				D−	D+	C−	C+
Procyanidin trimer (possibly C2)	10.16	280	867, *291*	13.69	16.00	10.10	10.00
Catechin	12.67	280	291	-	-	9.45	8.73
Procyanidin trimer C1	14.33	280	867, *291*	-	-	6.85	5.38

* The amount of each identified compound in 100 g initial sample (tissue) was expressed in catechin equivalents where duodenum sample from piglets fed with the regular diet = D−, duodenum sample from piglets fed with 5% GP diet = D+, colon sample from piglets fed with the regular diet = colon C− and colon sample from piglets fed with 5% GP diet = C+ samples.
